# Synthesis of Bis-Chalcones and Evaluation of Its Effect on Peroxide-Induced Cell Death and Lipopolysaccharide-Induced Cytokine Production

**DOI:** 10.3390/molecules28176354

**Published:** 2023-08-30

**Authors:** Alby Tom, Jisha Jacob, Manoj Mathews, Rajakrishnan Rajagopal, Ahmed Alfarhan, Damia Barcelo, Arunaksharan Narayanankutty

**Affiliations:** 1 Division of Cell and Molecular Biology, PG and Research Department of Zoology, St. Joseph’s College Devagiri (Autonomous), Calicut 673008, Kerala, India; albytom333@gmail.com; 2 Molecular Microbial Ecology Lab, PG and Research Department of Zoology, St. Joseph’s College Devagiri (Autonomous), Calicut 680555, Kerala, India; jishajacob@devagiricollege.org; 3 PG and Research Department of Chemistry, St. Joseph’s College Devagiri (Autonomous), Calicut 680555, Kerala, India; manojmathews@devagiricollege.org; 4 Department of Botany and Microbiology, College of Science, King Saud University, P.O. Box 2455, Riyadh 11451, Saudi Arabia; rrajagopal@ksu.edu.sa (R.R.); alfarhan@ksu.edu.sa (A.A.); 5 Water and Soil Research Group, Department of Environmental Chemistry, Idaea-Csic, Jordi Girona 18-26, 08034 Barcelona, Spain; damia.barcelo@idaea.csic.es

**Keywords:** bis-chalcones, antioxidant activity, cytoprotection, peroxide toxicity, anti-inflammatory activity

## Abstract

Plant secondary metabolites are important sources of biologically active compounds with wide pharmacological potentials. Among the different classes, the chalcones form integral pharmacologically active agents. Natural chalcones and bis-chalcones exhibit high antioxidant and anti-inflammatory properties in various experiments. Studies are also underway to explore more biologically active bis-chalcones by chemical synthesis of these compounds. In this study, the effects of six synthetic bis-chalcones were evaluated in intestinal epithelial cells (IEC-6); further, the anti-inflammatory potentials were studied in lipopolysaccharide-induced cytokine production in macrophages. The synthesized bis-chalcones differ from each other first of all by the nature of the aromatic cores (functional group substitution, and their position) and by the size of a central alicycle. The exposure of IEC-6 cells to peroxide radicals reduced the cell viability; however, pre-treatment with the bis-chalcones improved the cell viability in these cells. The mechanism of action was observed to be the increased levels of glutathione and antioxidant enzyme activities. Further, these bis-chalcones also inhibited the LPS-stimulation-induced inflammatory cytokine production in RAW 264.7 macrophages. Overall, the present study indicated the cytoprotective and anti-inflammatory abilities of synthetic bis-chalcones.

## 1. Introduction

Colorectal cancers (CRCs) are one among the major concerns of healthcare systems in developed and developing countries. Colorectal cancer is the second most common cancer in the United States and it also accounts for approximately 52,550 deaths in the country [1]. In addition, the report emphasized the increase in the occurrence of CRCs among those younger than 50 years of age [1,2,3]. In the Indian population, the incidence of CRC is much lower with an age-standardized ratio of 7.2 per 100,000; however, it should be noted that the country accounts for a significantly lower 5-year survival rate in CRC with respect to global standards [4,5]. Oxidative stress is a key event in the onset and progression of intestinal disorders including colorectal cancers [6,7]. The initial events of oxidative stress begin with the imbalance in the production of reactive radical moieties in the cells and also with the scavenging of these reactive molecules [8]. These free radicals include the oxygen, nitrogen and lipid-derived radicals and among these, the predominant ones are the peroxides [9]. The peroxide radicals are commonly formed in the intestinal epithelial cells and are known to induce genomic instability and cell death [10]. The increased levels of peroxides are also shown to increase the expression of angiogenic factors in CRCs [11]. Increased oxidative damage is also associated with the metastatic phenotype in CRCs [12]. The intestinal peroxide radicals are also contributors to inducing ulcerative colitis by driving inflammatory events in the colon [13]. The inflammatory cascades are also associated with the promotion of various cancers including colorectal cancer [14,15]. The inflammatory cytokines and other inflammatory mediators are reported to be elevated in the advanced stages of colorectal cancers. Similarly, increased levels of inflammatory NF-κB are associated with the metastatic phenotype of colorectal cancer cells [16]. Tumor-associated macrophages are also involved in the induction of the inflammatory tumor microenvironment and subsequent progression of colorectal cancers [17]. Hence, the prevention of oxidative stress and inflammation forms a strategic response in the prevention and onset of colorectal cancers.

Natural products, especially those of plant origin, are important radical scavengers and anti-inflammatory molecules [18]. Among the different classes, the polyphenols, flavonoids, anthocyanins, terpenoids, anthraquinones, etc. are important compounds. Among these, chalcones and bis-chalcones are important compounds which belong to the flavonoid family. Chalcones are the unsaturated α, β-ketones with terminal aromatic fragments [19]. The Asteraceae, Leguminosae and Moraceae members of plants contain a large quantity of natural chalcones [20,21]. Several biological and pharmacological properties are attributed to these chalcones. The compounds are reported to inhibit the oxidative radical formation in different in vitro and in vivo models [22,23]. Together with this, the compounds are reported to enhance antioxidant defense in animals [24,25]. It has also been reported that chalcones and bis-chalcones reduce cytokine production and nitric oxide synthase activity in macrophages [26,27]. It has further been reported that chalcones effectively regulate prostaglandin synthesis and NF-κB signaling and thereby reduce inflammation in the cellular milieu [28]. The yield of chalcones in plants is low and the purification process is difficult. However, synthesis of chalcones is possible and is an alternative for yielding high-purity chalcones within a short period of time [29,30]. 

**Figure 1:**
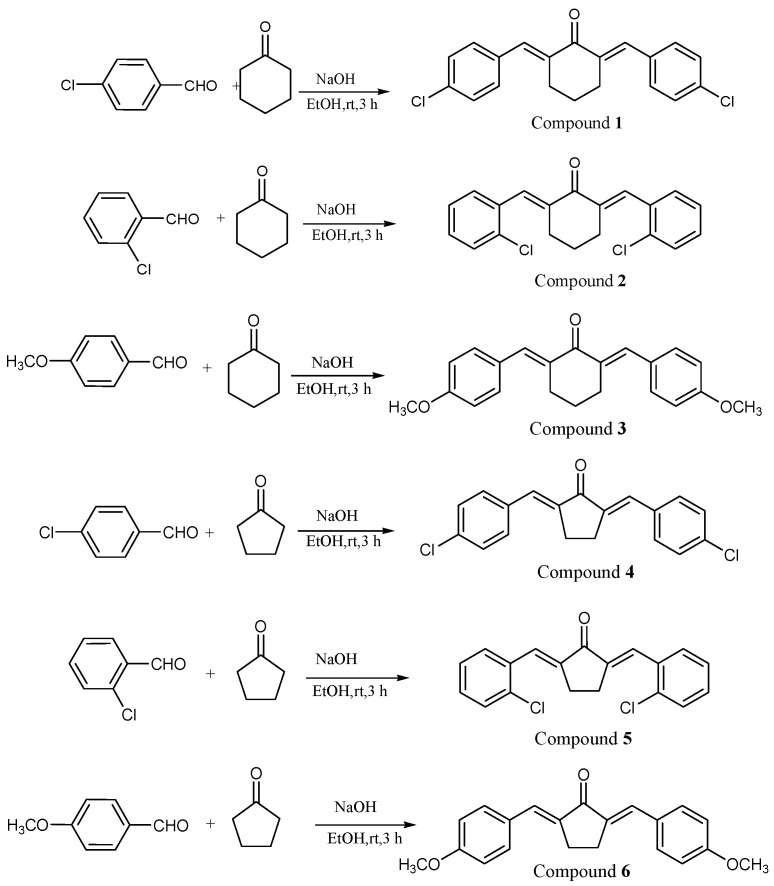
The synthesis and structure of different synthetic bis-chalcones.

Hence, the present study aimed to analyze the cytoprotective and antioxidant abilities of synthetic bis-chalcones (Figure 1) that were proven to have antioxidant properties in our previous study [31]. The present work focused on three compounds from the previous study, compounds **1**, **2** and **4**. In addition, we evaluated the anti-inflammatory properties of these compounds in LPS-stimulated macrophages.

## 2. Results and Discussion

### 2.1. Characterization of Compounds

Chalcones and bis-chalcones are important bio-organic compounds that are naturally occurring in plants [32]. The chalcones are synthesized in plants using chalcones synthase enzymes and they act as intermediates in the production of flavonoids [33]. They also perform integral roles as floral pigments, insect repellents, and antimicrobial and UV protective molecules in plants [34]. However, due to the difficulty in extraction and limited availability and yield from plants, the synthetic preparations are now considered to replace the natural chalcones and bis-chalcones. Compared to chalcones, bis-chalcones are less explored for their biological efficacies. In the present study, we synthesized six bis-chalcones and evaluated them for their cytoprotective and anti-inflammatory effects (the data of only three are shown, since compounds **3**, **4** and **5** had no activity).

Compound **1** was obtained as yellow crystals at 66% yield by mixed solvent recrystallization. The physico-chemical properties were as follows; MP: 220–225 °C. R_f_ = 0.66 in 20% EtOAc—Hexane. The FTIR spectra represented peaks (cm^−1^) as 3058, 2963, 1670, 1605, 1575, 1488, 1401, 1319, 1264, 1091, 977, 929, 835, 821, 798, and 524. The result of the NMR was: ^1^H NMR (500 MHz, CDCl_3_): δ ppm 7.7(s, 2H, Olefinic-H), 7.35 (t, 8H, Ar-H), 2.86–2.84 (m, 4H, 2xCH_2_), and 1.81–1.74 (m, 2H, 1xCH_2_).

Compound **2** was obtained as yellow crystals by mixed solvent recrystallization at 90.6% yield. The physico-chemical properties were as follows; MP: 220–225 °C. R_f_ = 0.4883 in 20% EtOAc—Hexane. The FTIR spectra represented peaks (cm^−1^) as 3070, 2970, 1663, 1601, 1575, 1467, 1432, 1260, 1169, 1145, 1119.5, 977, 929, 835, 821, 766, and 735. The result of the NMR was: ^1^H NMR (500 MHz, CDCl_3_): δ ppm 7.90 (s, 2H, Olefinic-H), 7.43–7.41 (m, 2H, Ar-H), 7.33–7.31 (m, 2H, Ar-H), 7.28–7.25 (m, 4H, Ar-H), 2.75 (t, 4H, J = 5 Hz, 2xCH_2_), and 1.34 (m, 2H, 1xCH_2_).

Compound **3** was obtained as yellow crystals by mixed solvent recrystallization at 48.8% yield. R_f_ = 0.73 in 20% EtOAc—Hexane. The FTIR spectra represented peaks (cm^−1^) as 2963, 2916, 2843, 2051, 1915, 1696, 1616, 1597, 1508, 1438, 1418, 1365, 1305, 1253, 1171, 1119, 1029, 987, 929, 835, 819, 689, 607, 536, and 517. The result of the NMR was: ^1^H NMR (500 MHz, CDCl_3_): δ ppm 7.76 (s, 2H,Olefinic-H), 7.46–7.44 (m,4H, Ar-H), 6.94–6.92 (m, 4H, Ar-H), 3.83 (s, 6H, 2 ×−OCH_3_), 1.8–1.6 (m, 2H, 1xCH_2_), and 2.92–2.89 (m, 4H, 2xCH_2_).

Compound **4** was obtained as yellow crystals by mixed solvent recrystallization at 49.4% yield. The physico-chemical properties were as follows: R_f_ = 0.58 in 20%. EtOAc—Hexane. The FTIR spectra represented peaks (cm^−1^) as 3378, 2919, 2366, 2342, 1913, 1694, 1621, 1607, 1584, 1556, 1489, 1404, 1306, 1278, 1253, 1178, 1106, 1092, 1009, 985, 929, 833, 820, 729, 685, 611, and 520. The result of the NMR was: ^1^H NMR (500 MHz, CDCl_3_): δ ppm 7.53–7.40 (m, 6 H, J = 8.5Hz, 2H (Olefinic-H), 4H (Ar-H), 7.34 (d, 4H, J = 8.5 Hz, Ar-H), and 3.01 (s, 4H, 2xCH_2_).

Compound **5** was obtained as yellow crystals by mixed solvent recrystallization at 69.5% yield. The physico-chemical properties were as follows: R_f_ = 0.51 in 20% EtOAc—Hexane. The FTIR spectra represented peaks (cm^−1^) as 3069, 2912, 2361, 1687, 1620, 1599, 1587, 1560, 1465, 1449, 1431, 1277, 1239, 1182, 1155, 1124, 1038, 987, 941, 759, 751, 726, 688, 622, 544, and 532. ^1^H NMR (500 MHz, CDCl_3_): δ ppm 7.90 (s, 2H, Olefinic-H), 7.53–7.42 (m, 4H, Ar-H), 7.30–7.27 (d, 4H, J = 1.5 Hz, Ar-H), and 2.97 (s, 4H, 2 × CH_2_). The result of the NMR was: ^13^C NMR (500 MHz, CDCl_3_): δ ppm 195.57, 139. 36, 136.13, 133.91, 130.30, 130.19, 130.13, 126.71, and 26.61.

Compound **6** was obtained as yellow crystals by mixed solvent recrystallization at 49.7% yield. The physico-chemical properties were as follows: R_f_ = 0.64 in 20% EtOAc—Hexane. The FTIR spectra represented peaks (cm^−1^) as 3069, 2963, 2937, 2913, 2846, 2057, 2007, 1696, 1616, 1597, 1508, 1439, 1365, 1306, 1252, 1171, 1029, 929, 835, 819, 694, 608, 536, and 517. The result of NMR was: ^1^H NMR (500 MHz, CDCl_3_): δ ppm 7.57–7.55 (m, 6H, Ar-H), 6.96–6.95 (m, 4H, Ar-H), 3.85 (s, 6H, 2 × OCH_3_), and 3.06 (s, 4H, 2xCH_2_). 

The characterization details of these chalcones using FT-IR and NMR spectroscopy are included in Supplementary Figures S1–S9. In Supplementary, the synthesis protocol of all six bis-chalcones has been included. (Characterization details of compounds **1**, **2** and **4** were included in the Supplementary Files because other compounds did not exhibit any activity.)

### 2.2. Cytotoxicity of Peroxide Radicals and Bio-Safe Concentration of Bis-Chalcones

**Figure 2:**
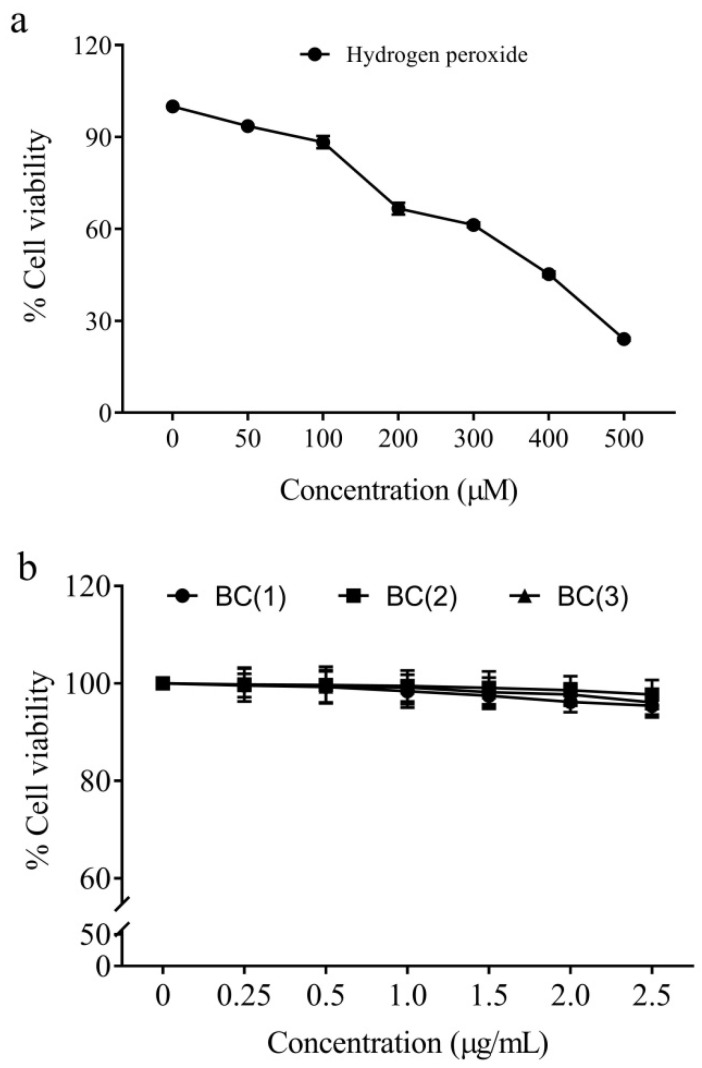
Cytotoxicity of hydrogen peroxide (**a**) and biologically safer concentration of synthetic bis-chalcones (**b**).

The cytotoxicity analysis by MTT assay revealed that the IC50 value of hydrogen peroxide was 391.8 ± 4.6 µM. Hence, the study opted for 400 µM as the test concentration of peroxide radicals to induce cytotoxicity in the IEC-6 cells (Figure 2).

The cytotoxicity evaluation of synthetic bis-chalcones (data not included) revealed that the IC50 values of the compounds were above 250 µg/mL (Figure 2). Hence, the biological safety analysis was limited to a concentration of 2.5 µg/mL; among these, the concentrations 0.5 and 1.0 µg/mL had no significant toxic effects on the cells and therefore those doses were opted for further cytoprotective studies.

### 2.3. Cytoprotective Effect of Bis-Chalcones

**Figure 3:**
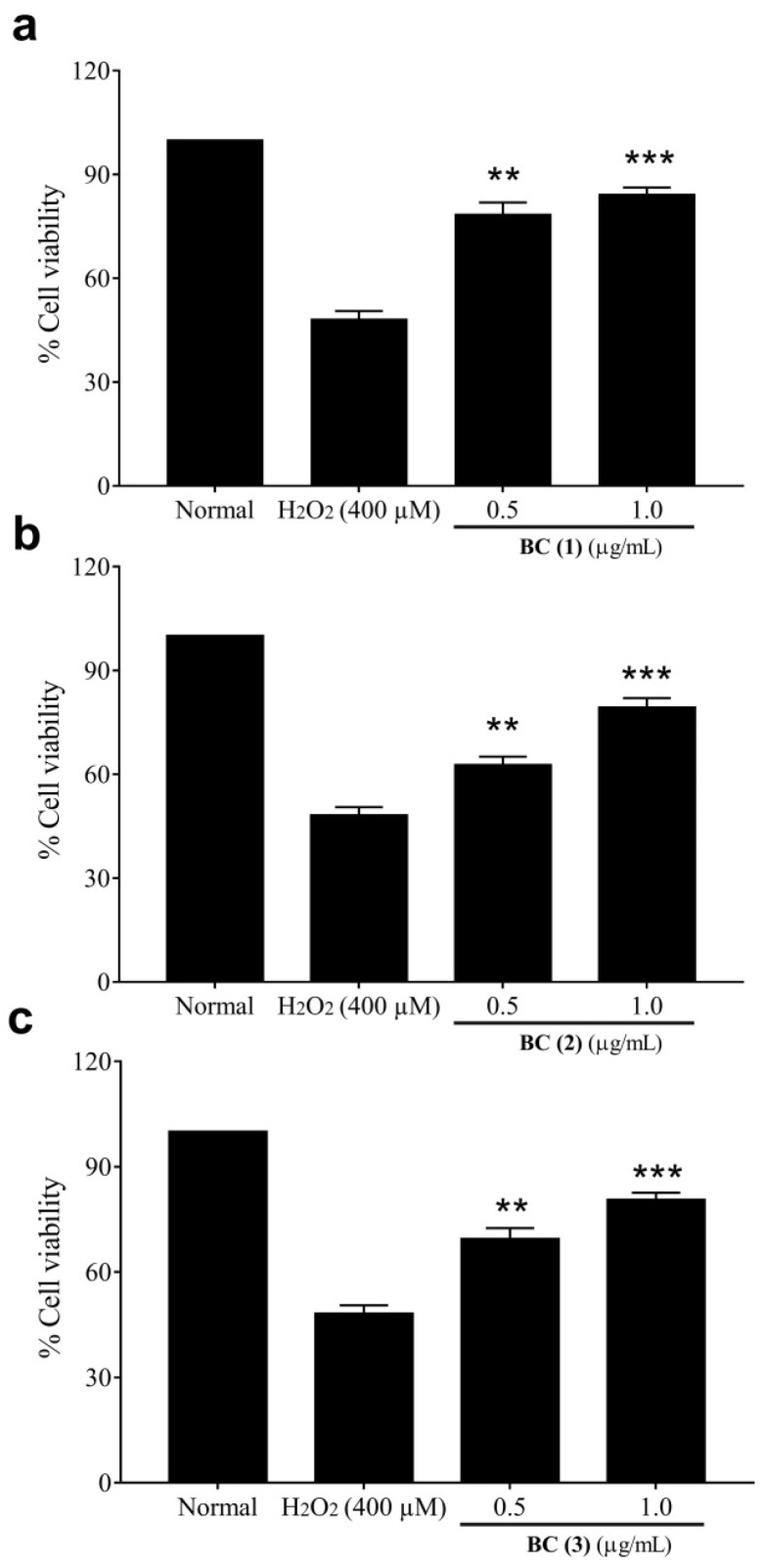
Protective effect of synthetic bischalcones BC (**1**) (**a**), BC (**2**) (**b**), BC (**3**) (**c**) against hydrogen peroxide-induced cell death in intestinal epithelial cells. (** indicate *p* < 0.01 and *** indicate *p* < 0.001).

The cytoprotective effect was analyzed against peroxide radicals by pre-incubating the cells with different doses of bis-chalcones for 2 h and exposing them to the radicals for 24 h. The untreated normal IEC-6 cells were considered to be 100% viable and the exposure of these cells to peroxide radicals reduced the net cell viability to 48.2 ± 2.3% (Figure 3). Increased peroxide radicals in cells are also shown to reduce the cell viability by inducing apoptotic cell death mediated through lipid peroxidation and DNA damage [35,36]. It is therefore possible that the increased cell death in peroxide exposure may be due to the apoptotic cell death in colon cells. However, pre-treatment with compound **1** increased the cell viability to 78.5 ± 3.4 and 84.3 ± 1.9% at the respective doses of 0.5 and 1.0 µg/mL. Likewise, the treatment with compound **2** was found to reduce the toxic insults of peroxide radicals and thereby increase cell viability to 62.7 ± 2.4 and 79.3 ± 2.7% at the respective doses of 0.5 and 1.0 µg/mL. Compound **3** also improved the reduction in cell viability induced by the radical to 69.4 ± 3.1 and 80.6 ± 2.0% at 0.5 and 1.0 µg/mL pre-treatment. However, compounds **3**, **5** and **6** had no protective effect and therefore are not shown in the present results. It is thus possible that compounds **1**, **2** and **4** may have been involved in the free radical scavenging and improvement in the cellular antioxidant defense and subsequently increased the cell viability.

The level of reduced glutathione content in the normal IEC-6 cells was estimated to be 4.68 ± 0.22 µmoles/mg protein. Upon exposure to the hydrogen peroxide radicals at a concentration of (400 µM) for 24 h, the level of reduced glutathione was significantly brought down to 2.31 ± 0.24 µmoles/mg protein (*p* < 0.05). It has been previously reported that increased peroxide radicals often tend to reduce the cellular glutathione pool [37]. Glutathione is the central antioxidant and its depletion is essential for the induction of apoptotic death in cells [38,39]; hence, the increased cell death in peroxide-exposed cells may be attributed to the glutathione depletion events.

Compared to the control cells, the treatment with compound **1** [BC(**1**)] significantly increased the cellular-reduced glutathione pool to 3.07 ± 0.21 (*p* < 0.05) and 3.78 ± 0.35 µmoles/mg protein (*p* < 0.01) at their respective doses of 0.5 µg/mL and 1.0 µg/mL. In compound **2** [BC(**2**)], the level of glutathione was increased to 2.94 ± 0.23 and 3.15 ± 0.18 µmoles/mg protein. In BC(**3**) treated cells, the glutathione content was elevated to 3.13 ± 0.30 and 3.85 ± 0.41 µmoles/mg protein. Hence, it is clear that treatment with bis-chalcones increased the cellular pool of reduced glutathione. A previous study by Kachadourian et al. [40] indicated the possible potential of chalcones as inducers of glutathione biosynthesis; hence, it is also possible that the treated chalcones may have induced glutathione biosynthesis in the cell and subsequently increased the cellular GSH levels (Table 1).

Table 1 represents the results of various treatment regimens on the catalase activity in IEC-6 cells. The normal IEC-6 cells exhibited a catalase activity of 47.64 ± 3.7 U/mg protein. However, exposure to the peroxide radicals over 24 h resulted in a shoot in catalase activity (88.19 ± 4.3 U/mg protein). The catalase enzyme plays an important role in the degradation of peroxide radicals to yield water and therefore detoxification [41]. In addition, the increased level of peroxide exposure has also been reported to increase the activity of catalase in cells [42]. Therefore, it may be possible that the exposure of cells to peroxide radicals resulted in an increase in catalase activity. Interestingly, the cell pre-treated with compound **1** [BC(**1**)] (at 0.5 µg/mL and 1.0 µg/mL) showed a significant reduction in the activity of the catalase enzyme (65.62 ± 3.4 and 50.04 ± 4.2 U/mg protein). Furthermore, the other compounds also indicated a similar reduction in the catalase activities to 72.11 ± 2.3 and 59.15 ± 6.1 U/mg protein ((BC(**2**)) as well as 70.55 ± 4.8, and 61.82 ± 6.4 U/mg protein (BC(**3**)). The restoration of catalase activity to near normal possibly indicates the protective effect of synthetic bis-chalcones on peroxide toxicity.

Glutathione peroxidase is another cellular enzyme associated with peroxide neutralization [43]; the glutathione-derived hydrogen moieties are utilized by this enzyme for the detoxification of the peroxide radicals [44]. The GPx is also important during excessive peroxide-induced inactivation of the catalase; under such conditions, the glutathione peroxide takes the central responsibility of the redox balance in cells [45]. In the present study, the GPx activity of normal cells was 65.65 ± 3.94 U/mg protein. Peroxide radical exposure resulted in an elevation in the GPx activity to 103.10 ± 4.82 U/mg protein (*p* < 0.001). On the contrary, the pre-treatment with BC(**1**), BC(**2**), and BC(**3**) brought down the peroxide-induced elevation in the GPx activity (Table 1). Hence, it is possible that the acute exposure to peroxide may have resulted in the elevation in GPx activity; however, pre-treatment with chalcones may have partially scavenged the radicals and thereby resulted in the reduction in GPx activity.

The altered antioxidant defense is often expressed as increased lipid peroxidation products [46]; TBARS is considered to be an important marker of the extent of lipid peroxidation [47]. In the present study, the level of TBARS in normal cells was 1.75 ± 0.34 nmoles/mg protein. The peroxide exposure resulted in a significant increase in the TBARS level (6.55 ± 0.45 nmoles/mg protein). However, pre-treatment with BC(**1**) reduced the levels to 4.59 ± 0.21 and 3.25 ± 0.42 nmoles/mg protein; the level in BC(**2**) treatment was found to be 4.76 ± 0.40 and 4.05 ± 0.51 nmoles/mg protein. However, the BC(**3**) was less effective in preventing TBARS formation and the level was estimated to be 5.01 ± 0.17 and 4.11 ± 0.38 nmoles/mg protein (Table 1).

### 2.4. Anti-Inflammatory Effects of Bis-Chalcones

Inflammation plays a crucial role in the oncogenic transformation of colorectal epithelial cells [48,49]. Hence, the management of inflammatory insults also becomes important to prevent the colon carcinogenesis process. Being an integral ingredient of functional foods, the chalcones are one among the promising anticancer dietary agents [50]. In the present study, LPS-mediated inflammation was used as a model; LPS induces inflammatory cytokine release from macrophages by stimulating toll-like receptor mediated signaling [51,52].

Likewise, the level of IL-1β in untreated cells was 54.5 ± 2.9 pg/mg protein. Exposure of RAW 264.7 cells to LPS resulted in the increased secretion of IL-1β (503.2 ± 12.3 pg/mg protein). On the contrary, the pre-treatment with BC(**1**) at 0.5 and 1.0 µg/mL resulted in reduced IL-1β levels to 407.8 ± 15.6 and 298.4 ± 12.4 pg/mg protein. Furthermore, pre-treatment with BC(**2**) at the same concentrations reduced the level of IL-1β to 421.8 ± 14.5 and 365.7 ± 15.5 pg/mg protein. Likewise, pre-treatment with BC(**3**) reduced IL-1β levels to 434.5 ± 10.9 and 389.4 ± 16.2 pg/mg protein at 0.5 and 1.0 µg/mL. The role of IL-1β in colon cancer is evident as it promoted the proliferation potential of the cells and also triggered epithelial to mesenchymal transition in these cells [53,54]. Hence, inhibition of IL-1β secretion is therefore beneficial in intestinal conditions.

IL-6 is another pro-inflammatory cytokine that triggers transformation in colon epithelial cells by triggering STAT3 signaling cascades [55,56]. In untreated cells, the level of IL-6 was estimated to be 103.4 ± 10.2 pg/mg protein. However, LPS stimulation increased the secretion of IL-6 and thereby the level was elevated to 1185.2 ± 24.6 pg/mg protein. Treatment with BC(**1**) reduced the IL-6 levels to 851.1 ± 20.6 and 756.1 ± 22.4 pg/mg protein at its low and high doses. Likewise, pre-treatment with BC(**2**) (927.5 ± 27.3 and 835.1 ± 17.2 pg/mg protein) and BC(**3**) (964.7 ± 19.5 and 876.1 ± 27.4 pg/mg protein) effectively inhibited the IL-6 production in macrophages.

Untreated cells had a TNF-α level of 259.4 ± 10.9 pg/mg protein, which was increased upon exposure to LPS (1635.0 ± 22.5 pg/mg protein). On the contrary, pre-treatment with BC(**1**), BC(**2**) and BC(**3**) at their low (0.5 µg/mL) and high (1.0 µg/mL) doses inhibited the production of TNF-α levels (Table 2). The TNF-α levels are crucial for the survival and proliferation of various cancer cells including colon [57]. The cytokine is also important in the progression events of colon cancer such as metastasis and stemness [58]. Numerous studies have indicated the potential of various chalcones against the production of inflammatory cytokines [59,60].

The untreated RAW 264.7 cells had a nitric oxide level of 8.5 ± 0.7 µM/mg protein. However, exposure to LPS stimulated the production of NO and the level was elevated to 67.8 ± 2.7 µM/mg protein. Treatment with BC(**1**) reduced the nitric oxide level in the cells to 39.5 ± 1.2 and 26.5 ± 1.4 µM/mg protein at the low and high doses, respectively. Likewise, pre-treatment with BC(**2**) brought down the cellular NO levels to 51.0 ± 0.5 and 39.2 ± 1.1 µM/mg protein. The nitric oxide level in the BC(**3**) pre-treated cells (0.5 µg/mL and 1.0 µg/mL) was found to be 46.7 ± 0.8 and 31.2 ± 1.8 µM/mg protein.

Overall, the results of the present study confirm the cytoprotective and anti-inflammatory potentials of the three synthetic bis-chalcones. However, further studies are necessary to study the molecular mechanism of action of these bis-chalcones, especially on the Nrf2/ARE axis of antioxidant defense.

## 3. Materials and Methods

### 3.1. Chemicals, Cells and Media

The chemicals were sourced from Sigma-Aldrich (St. Louis, MO, USA) and all the materials obtained were of analytical grade. The cells were procured from NCCS, Pune, India, and maintained under standard conditions in RPMI-1640 media (containing 10% FBS).

### 3.2. Synthesis and Characterization of Bis-Chalcones

The bis-chalcones were synthesized using cyclohexanone and cyclopentanone as core compounds. The detailed synthesis methods are illustrated in Supplementary Figures S1–S9. The compounds were previously synthesized as per our own publication [31].

### 3.3. Cytotoxicity Analysis of Peroxides and Biologically Safe Concentration of Bis-Chalcones

The non-cancer cell line IEC-6 (intestinal epithelial cells) and RAW 264.7 cells were procured from the National Centre for Cell Science and maintained in RPMI-1640 and DMEM media. The IEC-6 cells and RAW 264.7 were then plated in 96-well plates at a seeding density of 1 × 10^6^ cells/mL. The cells were treated with different doses of various bis-chalcones (0–250 µg/mL) for 48 h. At the end of incubation, the media were replaced with fresh media containing 5 mg/mL MTT. The cell viability was determined according to the previous descriptions of Khanapure et al. [61]. Similarly, the IC50 value of the peroxide radical was also determined using hydrogen peroxide as a radical source.

### 3.4. Analysis of the Effect of Bis-Chalcones against Peroxide-Induced Damage in Cells

In order to assess the protective effect against the peroxide radicals, the cells were plated as described in Section 3.3. The cells were pre-treated for 2 h with two biologically safe concentrations of individual bis-chalcones. The cells were further exposed to peroxide radicals after incubation for another 24 h. A peroxide control and untreated normal cells were also maintained to determine the extent of protection as indicated by the improvement in cell viability. The cell viability of each treatment was determined using MTT assay as described previously in Section 3.3 and the percentage of protection was observed.

Furthermore, to assess the mechanism of protection, the cells were again exposed to similar experimental conditions in Petri dishes. The cells were collected by mechanical scrapers and lysed in Tris buffer. The lysate was centrifuged at 8000× *g* for 15 min in a refrigerated centrifuge to yield clear supernatant. The activity of enzymes such as catalase, glutathione peroxidase and levels of glutathione as well as thiobarbituric acid reactive substances were also estimated according to our previous protocols [62].

### 3.5. Effect of Bis-Chalcones on LPS-Stimulated Macrophages

The anti-inflammatory activity was estimated in LPS-primer macrophages. Briefly, the macrophages were cultured in DMEM media for 48 h. The actively dividing cells were again plated onto a 6-well-plate. After 24 h, the cell was pre-treated with different biosafe concentrations of various bis-chalcones. Subsequently, the cells were exposed to 1 mg/mL concentration of lipopolysaccharide. At the end of the incubation, the cell and media were collected and stored at −80 °C for the analysis of inflammatory cytokine and biochemical detection of the nitric oxide formed [63]. The cytokine levels were determined using ELISA kits from Peprotech. 

### 3.6. Statistical Analysis

The obtained results were processed in MS Office Excel and the statistical comparison was carried out using two-way ANOVA in a GraphPad Prism version 10.0 (La Jolla, CA, USA).

## 4. Conclusions

The study analyzed the cytoprotective and anti-inflammatory activity of six different synthetic bis-chalcones. Among those tested, three were found to be active as anti-inflammatory and cytoprotective agents; they include compounds **1**, **2** and **4**. The cytoprotective effect was efficiently modulated through the restoration of antioxidant enzyme activities and reduction of subsequent lipid peroxidation. The inhibition of inflammatory cytokine production is found to be the mechanism of anti-inflammatory activity. Overall, the synthetic bis-chalcones **1**, **2** and **4** are promising as possible therapeutic candidates against oxidative insult and inflammation. To be more specific, compound **2** was found to be more effective and seems to be promising as a pharmacological agent for future use. However, further studies are necessary on the toxicity aspects of these compounds in animals as well as confirmation of their protective effect in rat/mice models.

## Figures and Tables

**Table 1:** Changes in the cellular redox status in cells exposed to peroxide radicals and the effect of synthetic bis-chalcones.

Treatment	Catalase (U/mg Protein)	GSH (µmoles/mg Protein)	GPx (U/mg Protein)	TBARS (nmoles/mg Protein)
Normal	47.64 ± 3.7	4.68 ± 0.22	65.65 ± 3.94	1.75 ± 0.34
H_2_O_2_ (400 µM)	88.19 ± 4.3	2.31 ± 0.24	103.10 ± 4.82	6.55 ± 0.45
BC(**1**) 0.5 µg/mL	65.62 ± 3.4 **	3.07 ± 0.21 *	85.64 ± 4.12 **	4.59 ± 0.21 *
BC(**1**) 1.0 µg/mL	50.04 ± 4.2 ***	3.78 ± 0.35 **	75.05 ± 3.14 ***	3.25 ± 0.42 **
BC(**2**) 0.5 µg/mL	72.11 ± 2.3 *	2.94 ± 0.23 **	90.04 ± 2.15 *	4.76 ± 0.40 *
BC(**2**) 1.0 µg/mL	59.15 ± 6.1 ***	3.15 ± 0.18	84.11 ± 3.45 **	4.05 ± 0.51 **
BC(**3**) 0.5 µg/mL	70.55 ± 4.8 *	3.13 ± 0.30 *	80.17 ± 4.03 *	5.01 ± 0.17 *
BC(**3**) 1.0 µg/mL	61.82 ± 6.4 **	3.85 ± 0.41 **	70.52 ± 4.16 **	4.11 ± 0.38 **

BC(**1**)—Bis-chalcone **1** (compound **1**); BC(**2**)—Bis-chalcone **2** (compound **2**); BC(**3**)—Bis-chalcone **3** (compound **3**). The * indicates significant variation at *p* < 0.05, ** indicate *p* < 0.01 and *** indicate *p* < 0.001.

**Table 2:** Effect of synthetic bis-chalcones on LPS-stimulated cytokine release in RAW 264.7 cells.

	IL-1β (pg/mg Protein)	IL-6 (pg/mg Protein)	TNF-α (pg/mg Protein)	NO (µM/mg Protein)
Untreated	54.5 ± 2.9	103.4 ± 10.2	259.4 ± 10.9	8.5 ± 0.7
LPS	503.2 ± 12.3	1185.2 ± 24.6	1635.0 ± 22.5	67.8 ± 2.7
BC(**1**) 0.5 µg/mL	407.8 ± 15.6 *	851.1 ± 20.6 *	1367.0 ± 18.3 **	39.5 ± 1.2 *
BC(**1**) 1.0 µg/mL	298.4 ± 12.4 ***	756.1 ± 22.4 ***	1015.1 ± 18.6 ***	26.5 ± 1.4 ***
BC(**2**) 0.5 µg/mL	421.8 ± 14.5 *	927.5 ± 27.3 *	1475.8 ± 10.4 *	51.0 ± 0.5 ^ns^
BC(**2**) 1.0 µg/mL	365.7 ± 15.5 **	835.1 ± 17.2 **	1300.7 ± 33.4 **	39.2 ± 1.1 *
BC(**3**) 0.5 µg/mL	434.5 ± 10.9 *	964.7 ± 19.5 *	1404.1 ± 28.2 *	46.7 ± 0.8 *
BC(**3**) 1.0 µg/mL	389.4 ± 16.2 **	876.1 ± 27.4 **	1288.5 ± 16.3 **	31.2 ± 1.8 **

The values are represented as mean ± SD of three independent experiments, each carried in triplicate. (^ns^ indicates not significant; * indicates significant difference *p* < 0.05; ** indicate significant difference *p* < 0.01 and *** indicate significant difference *p* < 0.001).

## Data Availability

The data may be shared upon valid request.

## Supplementary Materials

The following supporting information can be downloaded at: https://www.mdpi.com/article/10.3390/molecules28176354/s1. Figure S1: Synthesis of Compound **1**; Figure S2: FT-IR spectrum of compound **1**; Supplementary Figure S3: ^1^H NMR Spectrum of compound **1** in CDCl_3_; Figure S4: Synthesis of Compound **2**; Figure S5: FT-IR spectrum of compound **2**; Figure S6: ^1^H NMR Spectrum of compound **2** in CDCl_3_; Figure S7: Synthesis of Compound **4**; Figure S8: FT-IR spectrum of compound **4**; Figure S9: ^1^H NMR Spectrum of compound **4** in CDCl_3_.

## Sample Availability

Samples of the compounds are available from the authors.
