# Supplementary material for: Synthesis of Bis-Chalcones and Evaluation of Its Effect on Peroxide-Induced Cell Death and Lipopolysaccharide-Induced Cytokine Production
Source: Molecules. 2023 Aug 30;28(17):6354. doi: 10.3390/molecules28176354 (PMC10488834; doi:10.3390/molecules28176354)
Supplement: Supplementary file 1 [file molecules-28-06354-s001.zip › molecules-2561717-supplementary.pdf]

Synthesis of Compound 1

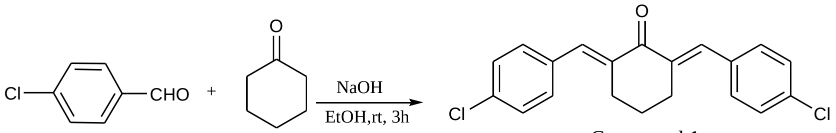

Compound **1**

Figure S1: Synthesis of Compound **1**

To a stirring solution of cyclohexanone (1 equivalent) and *p*-chlorobenzaldehyde (2 equivalent) in ethanol was added a solution of 10% NaOH in ethanol. The resulting solution was allowed to stir at room temperature. The reaction mixture was cooled in an ice bath, and the product was collected by suction filtration on the Buchner funnel. Mixed solvent recrystallization from DCM-EtOH afforded compound 1 as yellow crystals in 66% yield. MP: 220-225 ^{o}C. R_f =0.66 in 20% EtOAc – Hexane, IR (cm^{-1}): 3058, 2963, 1670, 1605, 1575, 1488, 1401, 1319, 1264, 1091, 977, 929, 835, 821, 798, 524.

^{1}H NMR (500 MHz, $CDCl_3$): ppm 7.7(s, 2H, Olefinic-H),7.35 (t, 8H, Ar-H), 2.86 - 2.84 (m, 4H, $2xCH_2$), 1.81-1.74 (m, 2H, $1xCH_2$).

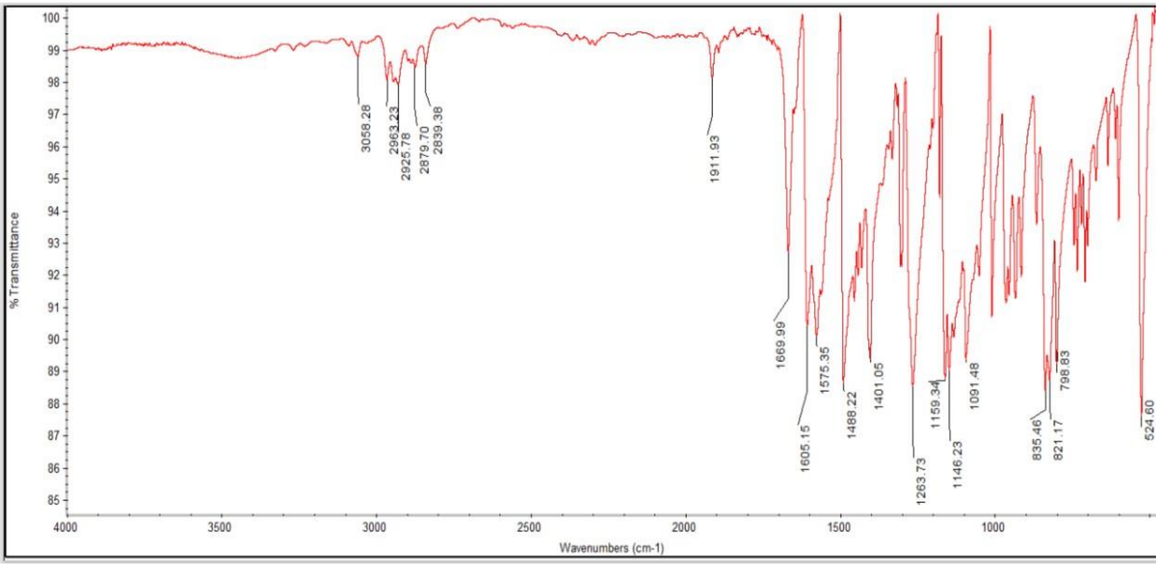

Figure S2: FT-IR spectrum of compound **1**

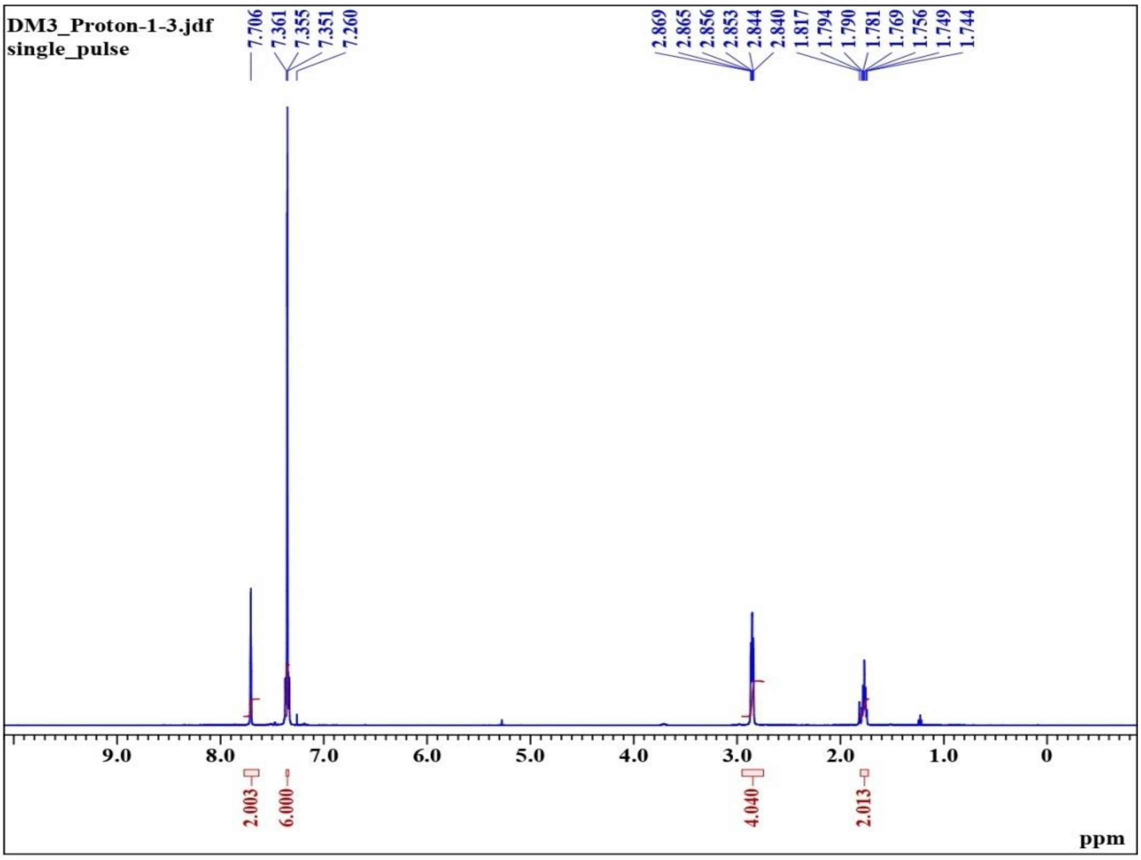

Figure S3: ^{1}H NMR Spectrum of compound **1** in $CDCl_3$

Synthesis of Compound 2

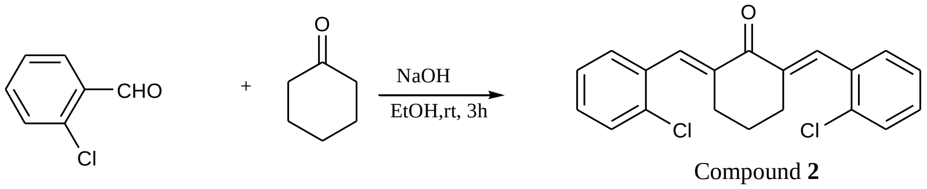

Compound **2**

Figure S4: Synthesis of Compound **2**

To a stirring solution of cyclohexanone (1 equivalent) and *o*-chlorobenzaldehyde (2 equivalent) in ethanol was added a solution of 10% NaOH in ethanol. The resulting solution was allowed to stir at room temperature. The reaction mixture was cooled in an ice bath, and the product was collected by suction filtration on Buchner funnel. A mixed solvent recrystallisation from DCM−EtOH afforded compound **2** as yellow crystals in 90.6 % yield. MP: 220-225 ^{o}C. R_f=0.4883 in 20% EtOAc – Hexane.

IR (cm^{-1}): 3070, 2970, 1663,1601, 1575, 1467, 1432, 1260, 1169, 1145, 1119.5, 977, 929, 835, 821, 766, 735.

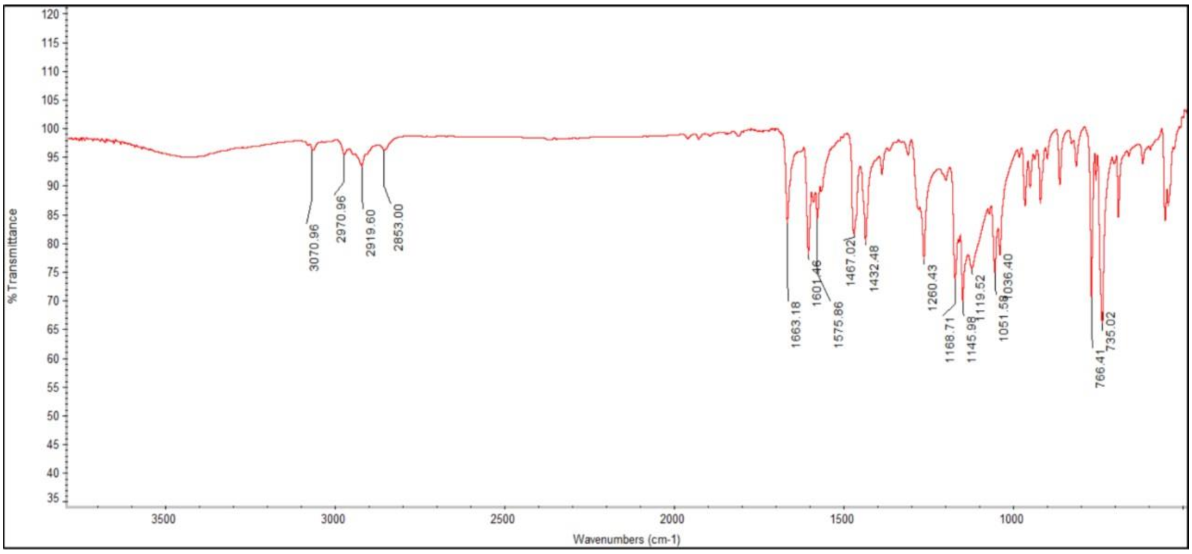

Figure S5: FT-IR spectrum of compound **2**

^{1}H NMR (500 MHz, $CDCl_3$): ppm 7.90 (s, 2H, Olefinic-H), 7.43-7.41(m, 2H, Ar-H), 7.33-7.31(m, 2H, Ar-H), 7.28-7.25(m, 4H, Ar-H), 2.75(t, 4H, J=5Hz, $2xCH_2$), 1.34 (m, 2H, $1xCH_2$).

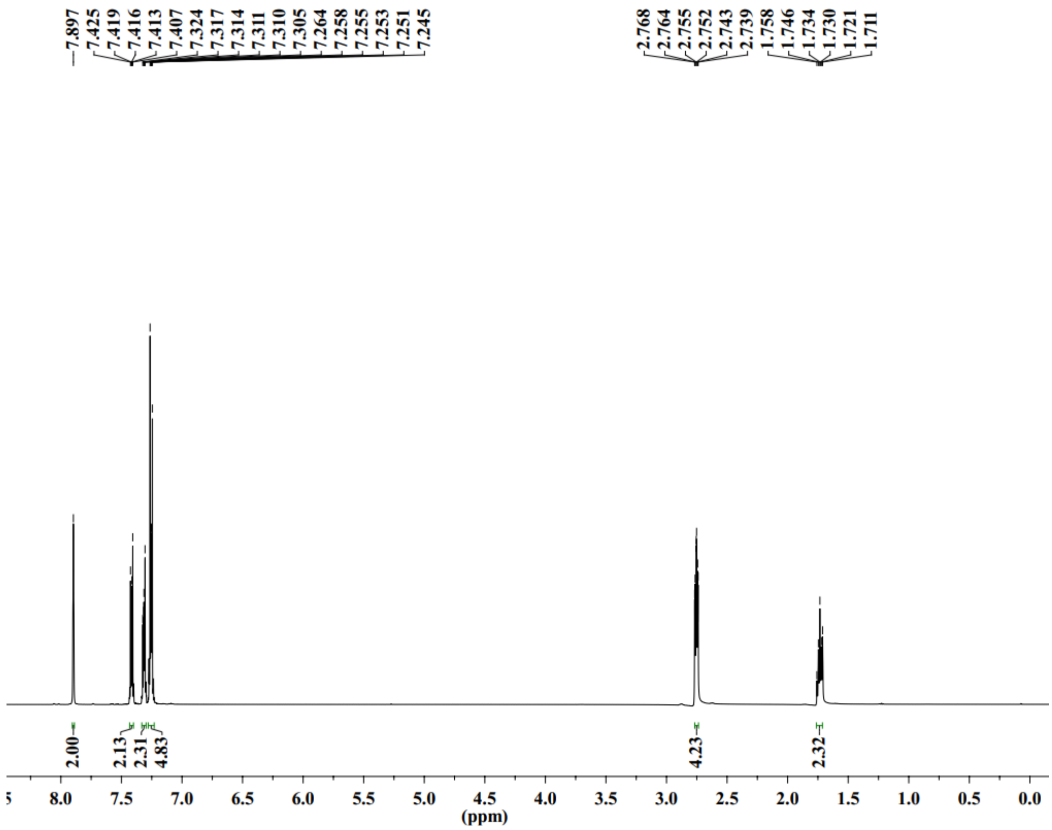

Figure S6: ^{1}H NMR Spectrum of compound **2** in $CDCl_3$

Synthesis of Compound 4

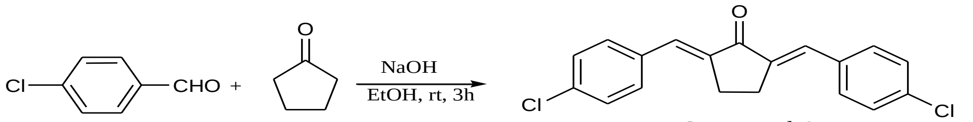

Compound **4**

Figure S7: Synthesis of Compound **4**

To a stirring solution of cyclopentanone (1 equivalent) and *p*-chlorobenzaldehyde (2 equivalent) in ethanol was added a solution of 10% NaOH in ethanol. The resulting solution was allowed to stir at room temperature. The reaction mixture was cooled in an ice bath, and the product was collected by suction filtration on the Buchner funnel. Mixed solvent recrystallization from DCM−EtOH afforded compound 4 as yellow crystals in 49.4% yield. R_f =0.58 in 20%. EtOAc – Hexane, IR(ν cm^{-1}) 3378, 2919, 2366, 2342, 1913, 1694, 1621, 1607, 1584, 1556, 1489, 1404, 1306, 1278, 1253, 1178, 1106, 1092, 1009, 985, 929, 833, 820, 729, 685, 611, 520. ^{1}H NMR (500 MHz, $CDCl_3$): ppm 7.53-7.40 (m, 6 H, J = 8.5Hz, 2H (Olefinic-H), 4H (Ar-H), 7.34 (d ,4H, J = 8.5 Hz, Ar-H), 3.01 (s, 4H, 2xCH_2).

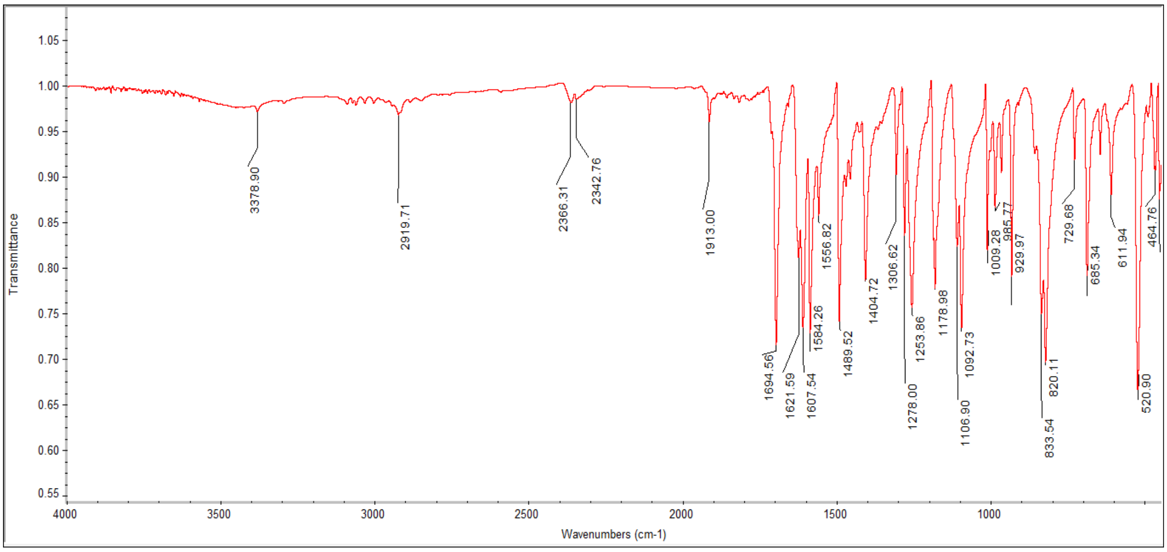

Figure S8: FT-IR spectrum of compound **4**

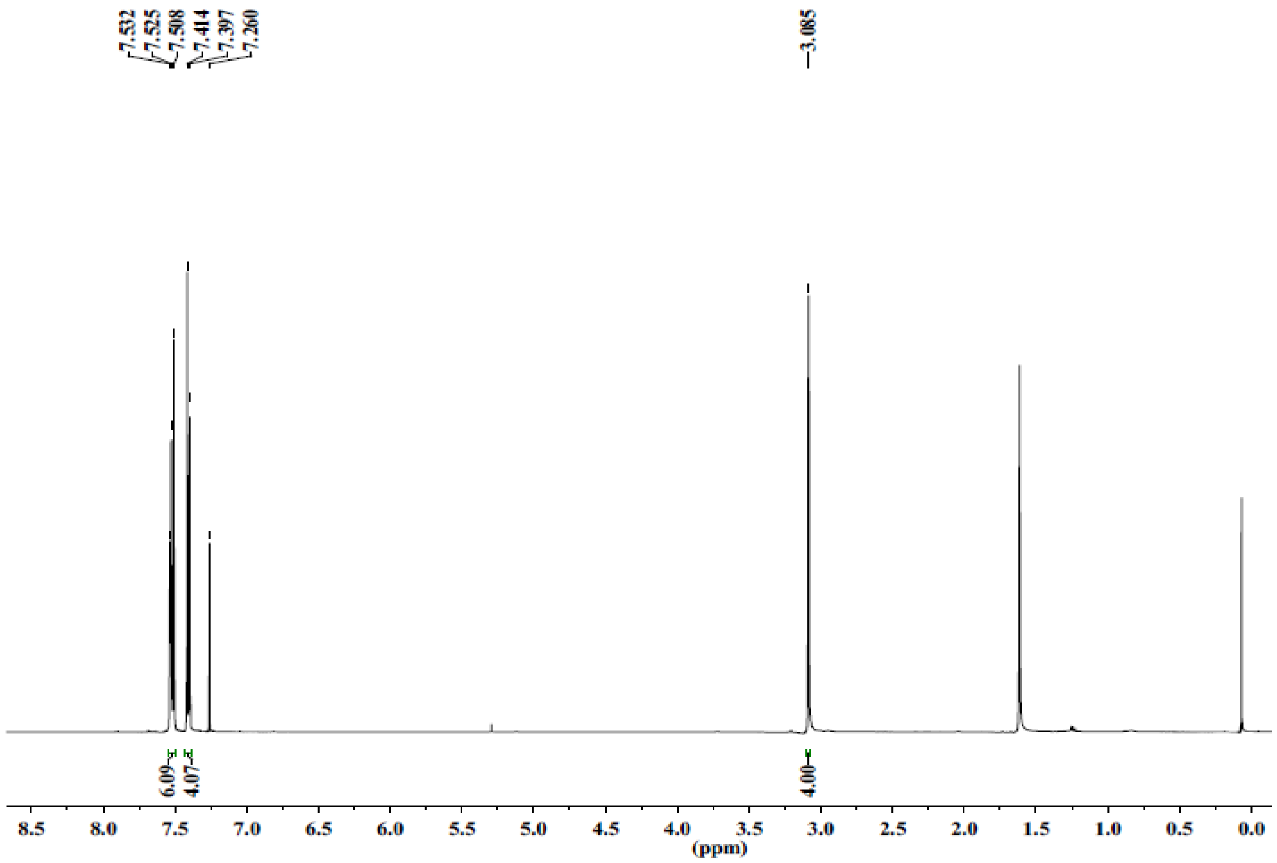

Figure S9: ^{1}H NMR Spectrum of compound **4** in $CDCl_3$.

In figure 1 in main text synthetic route for the target compounds is explained. The required chemicals such as ortho-chloro benzaldehyde, para-chloro benzaldehyde, para-methoxy benzaldehyde, cyclohexanone and cyclopentanone were obtained from Aldrich and used without further purification. Solvents and other reagents were obtained from local sources. The base-catalyzed Claisen–Schmidt condensation reaction of substituted benzaldehydes with cyclohexanone and cyclopentanone yielded the target compounds. In a typical reaction, the double mixed-aldol condensation reaction between a ketone and substituted benzaldehyde compound was carried out [16]. The ketone has α-hydrogens (on both sides) and thus can be deprotonated to give a nucleophilic enolate anion. The alkoxide produced is protonated by the solvent, giving a β-hydroxy ketone, which undergoes base-catalyzed dehydration. The elimination process is particularly fast in this case because the alkene is stabilized by conjugation not only to the carbonyl but also to the benzene. In this synthesis, two equivalents of the substituted benzaldehyde compound were used such that the aldol condensation could occur on both sides of the ketone. The aldehyde carbonyl is more reactive than that of the ketone and therefore reacts rapidly with the anion of the ketone to give a β-hydroxy ketone, which easily undergoes base-catalyzed dehydration. The molecular structures of all the target compounds were confirmed by standard spectroscopic methods of analysis. Detailed synthetic procedures for the compounds along with their characterization data are given as supplementary information. Instruments Used for the Study: IR spectra were recorded on a Nicolet iS5 Thermo Fischer Scientific FT-IR spectrometer (Waltham, MA, USA). The spectral positions are given in the wavenumber (cm−1) unit. 1H and 13C NMR spectra of the compounds in CDCl3 were recorded using Bruker AMX-400 (400 MHz) spectrometer (Billerica, MA, USA). For 1H NMR spectra, the chemical shifts (δ) are reported in parts per million (ppm) relative to tetramethylsilane (TMS) as an internal standard. Coupling constants (J) are given in Hz. The spectrophotometric measurements were taken using UV 1280 Shimadzu UV/Visible spectrophotometer (Kyoto, Japan).
